# Deep Sternal Wound Infection Caused by *Rhizopus* Species After Coronary Artery Bypass Graft

**DOI:** 10.1093/ofid/ofae302

**Published:** 2024-05-28

**Authors:** Cody A Cunningham, Thomas E Grys, Francis X Downey, Christopher F Saling, Robert Orenstein, Janis E Blair

**Affiliations:** Department of Internal Medicine, Mayo Clinic School of Graduate Medical Education, Scottsdale, Arizona, USA; Department of Pathology and Laboratory Medicine, Mayo Clinic Arizona, Phoenix, Arizona, USA; Department of Cardiothoracic Surgery, Mayo Clinic Arizona, Phoenix, Arizona, USA; Division of Infectious Disease, Mayo Clinic Arizona, Phoenix, Arizona, USA; Division of Infectious Disease, Mayo Clinic Arizona, Phoenix, Arizona, USA; Division of Infectious Disease, Mayo Clinic Arizona, Phoenix, Arizona, USA

**Keywords:** cardiac surgery, deep sternal wound infection, mediastinitis, Mucorales, *Rhizopus*

## Abstract

Deep sternal wound infection is a rare complication of cardiac surgery that is typically caused by skin resident flora, such as species of *Staphylococcus* and *Streptococcus*. Infections caused by fungi are less common and are generally caused by *Candida* species. Regardless of etiology, these infections are associated with significant morbidity and mortality. We present a case of postoperative mediastinitis that occurred following a 5-vessel coronary artery bypass graft and was caused by a filamentous fungus of the *Rhizopus* genus. The patient was treated with serial debridement, liposomal amphotericin B, and isavuconazonium and was discharged from the hospital in stable condition. Fungal mediastinitis is a rare entity, and clinicians must maintain a high level of suspicion to make the diagnosis. A fungal cause of postoperative mediastinitis should be considered in patients with negative bacterial cultures, uncontrolled diabetes, or current immunosuppression or those who present weeks after surgery with a subacute onset of symptoms.

Sternal wound infections are a rare complication of cardiac surgery. The risk of these infections varies by the type of procedure performed, with combined coronary artery bypass graft (CABG) and valve procedures having a higher risk than CABG or valve procedures alone [1]. Superficial wound infections (involving only the skin and subcutaneous tissue) complicate approximately 1.9% of these surgical procedures, whereas deep sternal wound infections (including mediastinitis) occur in about 0.7% of cases [2, 3]. These postoperative complications are associated with worsened morbidity and long-term mortality, even out to 4 years [2]. The incidence of sternal wound infections has declined in recent years due to improved prevention strategies, including chlorhexidine preparation of the skin sites, screening for methicillin-resistant *Staphylococcus aureus*, and treating nasal colonization preoperatively, and the use of perisurgical prophylactic antibiotics [4, 5].

Several risk factors have been identified that put patients at greater risk of a deep sternal wound infection. Impaired cardiac function, measured by patient symptoms (New York Heart Association class ≥3) or imaging (left ventricular ejection fraction <30%), appears to be an independent risk factor [2]. Other risk factors include obesity (body mass index ≥30 kg/m^2^), peripheral vascular disease, renal dysfunction, and insulin-dependent diabetes mellitus. From a surgical standpoint, utilization of the bilateral internal mammary arteries, the prevalence of which has been increasing [6], appears to increase the risk of deep sternal wound infections [7]. Additionally, a prolonged operative time, exceeding 300 minutes, is an independent risk factor [1].

Deep sternal wound infections are typically monomicrobial. In a large case series, *S aureus* was the most commonly identified cause, accounting for 60.1% of cases. Less commonly identified bacteria include gram-negative bacilli (16.5%), coagulase-negative staphylococcus (13%), and streptococci (4.7%) [8]. Fungi are a rare cause of deep sternal wound infections. *Candida* species accounted for 85% of cases identified in 1 case series of fungal mediastinitis [9]. Less common fungi identified in this series included the filamentous fungi *Trichosporon* spp (13%) and *Aspergillus fumigatus* (10%). Of note, no Mucorales species were identified in the case series.

We report a case of a deep sternal wound infection caused by a *Rhizopus* species that developed following a 5-vessel CABG procedure. The patient was treated with serial sternal wound debridements, isavuconazonium, and liposomal amphotericin B and was discharged from the hospital following a prolonged stay due to this infection.

## CASE PRESENTATION

A 76-year-old male with hypertension, hyperlipidemia, hypothyroidism, poorly controlled type 2 diabetes with insulin therapy, chronic kidney disease, and coronary artery disease was transferred to our tertiary care hospital for surgical management of a deep sternal wound infection. He was in his usual state of health until 5 weeks prior to admission to the outside hospital for a 5-vessel CABG (left internal mammary artery to left anterior descending, four saphenous vein grafts) and left atrial appendage closure. He tolerated the procedure well and was discharged home on postoperative day 3. He was initially recovering well, performing wound care as instructed and participating in home physical therapy. However, approximately 3 weeks after surgery he began to experience significant weakness, decreased oral intake, and insomnia. He also noted purulent drainage from his sternal wound and sought medical attention.

He underwent an evaluation by his cardiothoracic surgery team, which included a noncontrast computed tomography scan of the chest. This showed an evolving substernal hematoma or phlegmon and a left pleural effusion. He was therefore admitted to an outside hospital for further evaluation and management. Initial laboratory studies were notable for a white blood cell (WBC) count of 16 400/mm^3^ (reference range, 4500–11 000/mm^3^) and a lactate of 3.3 mmol/L (reference range, 0.4–2.0 mmol/L). Hemoglobin A_1c_ was 10.1% (reference range, 3.8%–5.7%) and blood glucose was 256 mg/dL (reference range, 70–110 mg/dL), although no evidence of diabetic ketoacidosis was seen. He was prescribed empirical antibiotic therapy with cefepime and vancomycin. He underwent 2 sternal irrigations and debridement procedures. Necrotic bone was debrided, resulting in a sizable sternal deficit. Surgical specimens were obtained and showed gram-positive cocci and WBCs on gram stain, but bacterial cultures had no growth. Following the second debridement, a wound vacuum was placed, and the patient was transferred to our hospital, as the outside facility did not have plastic and reconstructive surgery services.

**Figure 1.**
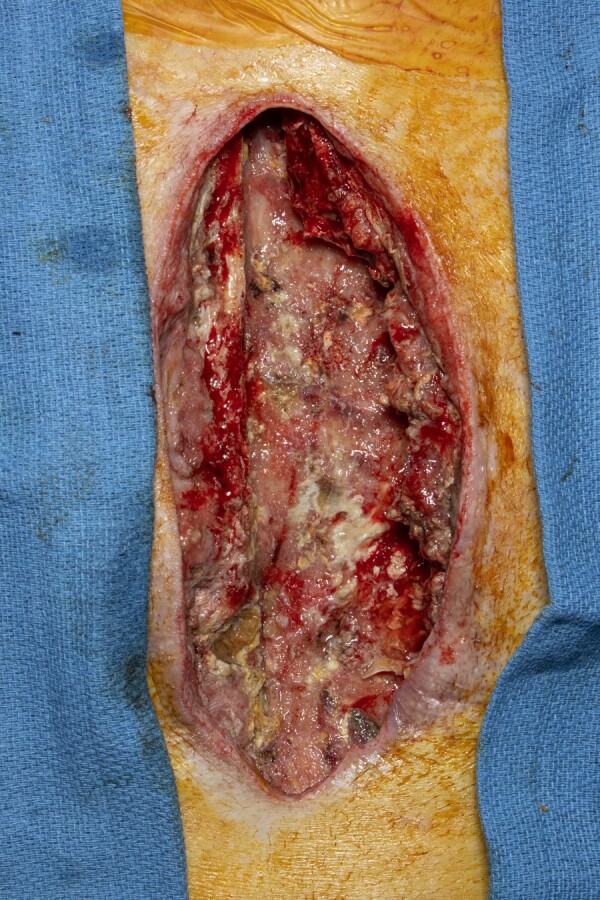
Gross appearance of the patient’s sternal wound prior to first surgical debridement shows devitalized tissue, most prominent at the left costal cartilage.

**Figure 2.**
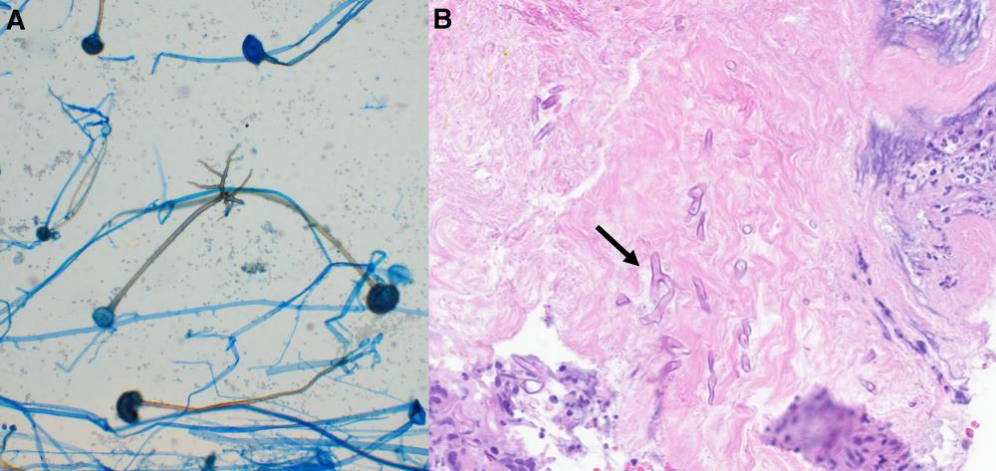
*A*, A tape preparation of a *Rhizopus* culture stained with lactophenol cotton blue. The morphology shows broad pauciseptate hyphae with rhizoids directly opposite sporangiophores. *B*, Hematoxylin and eosin staining of sternal bone from the patient's initial debridement at our hospital shows fragments of necrotic soft tissue, cartilage, and bone with acute inflammation. Present are scattered fungal organisms with broad ribbon-like hyphae, morphologically consistent with mucormycosis.

Upon arrival to our hospital, he was tachycardic (107 beats per minute), but the remainder of vital signs were within normal limits. Initial laboratory studies were notable for a hemoglobin level of 9.0 g/dL (reference range, 13.2–16.6 g/dL), WBC count of 13.2 × 10^9^/L (reference range, 3.4–9.6 × 10^9^/L), and platelet count of 504 × 10^9^/L (reference range, 135–317 × 10^9^/L). Hemoglobin A_1c_ was 9.7% (reference range, 4.2%–5.6%). The patient continued taking cefepime and vancomycin. On hospital day 2, the patient underwent a surgical debridement of the sternal wound. Gross inspection showed necrotic sternal tissue (Figure 1). Sternal bone was obtained for bacterial (aerobic and anaerobic), mycobacterial, and fungal cultures, and a new wound vacuum system was placed. Three days postprocedure, a fungal isolate consistent with a species of Mucorales was identified from the sternal bone and soft tissue cultures. He was initially prescribed isavuconazonium. The following day, the organism was identified as a *Rhizopus* species (Figure 2*A*). Four days after the initial operation, he underwent a second surgical debridement in which sternal bone was sent for pathology and showed fungal organisms consistent with *Rhizopus* invading necrotic bone and soft tissue (Figure 2*B*). Given the invasive nature of the patient's infection, he began taking liposomal amphotericin B dosed at 10 mg/kg/d. He ultimately had 7 debridements before uninfected margins were obtained. His sternal defect was closed on hospital day 21 with a rotational pectoral flap.

Following sternal closure, he underwent an additional 2 weeks of liposomal amphotericin B therapy (to ensure clinical stability) in addition to isavuconazonium. His diabetes was managed with long-acting insulin glargine (28 units daily) and short-acting insulin aspart (6 units with meals and for correction). The remainder of the patient's hospital course was complicated by an acute kidney injury (presumed due to the liposomal amphotericin) and prostatitis secondary to *Enterobacter cloacae*, which was treated with a course of levofloxacin. He was discharged on hospital day 36 with a 6-week course of levofloxacin and a tentative 6-month course of isavuconazonium, pending continued clinical improvement. The patient was seen 5 months after discharge and had no symptoms of infection despite self-discontinuing isavuconazonium after approximately 2 months. Magnetic resonance imaging of the chest will be obtained prior to restarting isavuconazonium.

## DISCUSSION AND CONCLUSIONS

Deep sternal wound infectious caused by fungi are rare [9]. A contemporary case series from France found that fungal mediastinitis occurred in 40 patients among 73 688 surgical procedures performed (0.05%). A significant fraction of these patients had received mechanical circulatory support (12.5%) or were prior orthotopic heart transplant recipients (30%). Eighty-five percent of these fungal infections were due to *Candida* species. No Mucorales infections were identified. Similar to the case presented here, the causative pathogen was identified several weeks following the initial surgery (range, 31–269 days depending on organism). The in-hospital mortality of fungal mediastinitis was high (58%) [9].

Mucorales are diverse group of molds that include *Mucor*, *Rhizopus*, *Rhizomucor*, and others [10]. The mode of transmission for Mucorales is believed to be predominantly via inhalation of spores from the environment. Direct inoculation by tissue trauma and introduction via needles and by contaminated adhesive products have also been described [10–13]. The mechanism by which the patient presented in this report was infected remains unknown. The patient did not have any notable environmental or occupational exposures that would place him at elevated risk. At present, this case appears to be isolated (not associated with a cluster of cases), arguing against faulty air handling or contaminated bandages as possible portals of entry.

Mucormycosis most commonly occurs as rhinocerebral, pulmonary, and skin infections. Regardless of the site of infection, tissue destruction occurs by angioinvasion with resultant tissue necrosis. One study identified 34 cases of osteoarticular infections caused by Mucorales between 1978 and 2014 [14]. Most cases were caused by *Rhizopus* (44.1%), *Apophysomyces* (11.7%), and *Mucor* (8.8%) species. Less common Mucorales included *Cunninghamella bertholletiae*, *Lichtheimia corymbifera*, and *Saksenaea vasiformis*. Only 1 case of sternal osteomyelitis was found [15]. This occurred in an immunocompetent 70-year-old male who was struck by the cut end of a tree he was trimming. The causative organism in this case was *Apophysomyces elegans*. Similar to the patient in our report, the patient underwent extensive surgical resection (sternectomy with rib resection) and treatment with amphotericin B.

Cooper et al reviewed 12 cases of mediastinal mucormycosis [16]. The overall mortality in this case series was 83%. Most cases were the result of fungal spread from a primary site, most often from the lungs. In this series, there was 1 case of postoperative mucormycosis infection, which involved a 21-year-old female with Loeys-Dietz syndrome type III who underwent an aortic root replacement with ascending aortic graft and bioprosthetic valve. Six weeks following surgery, the patient was diagnosed with an infectious pseudoaneurysm of the aorta, which ultimately grew *Rhizopus microsporus*. Similar to our case, this patient was treated with liposomal amphotericin B (5 mg/kg/d) in addition to caspofungin (for possible synergy). Amphotericin B was also administered by continuous mediastinal irrigation. Unfortunately, the patient had a pulseless electric activity arrest secondary to hemorrhagic shock and died on hospital day 20. Autopsy was notable for full-thickness necrosis of the aorta with abundant nonseptate hyphae.

The mainstay of treatment for Mucorales infections is surgical debridement and amphotericin B [17]. While current guidelines [18] for medical treatment recommend liposomal amphotericin B, to date no randomized controlled trials have been performed to establish amphotericin B as the superior first-line treatment. Indeed, case-control studies have suggested equivalency between patients treated with amphotericin B and isavuconazonium [19]. Additionally, posaconazole has been used as salvage therapy for patients who were intolerant of amphotericin B or whose disease progressed during amphotericin B therapy [20, 21]. Regardless of which medical regimen is chosen for initial therapy, early effective treatment and timely, aggressive surgical debridement are imperative. One study in patients with hematologic malignancies found that delayed administration of amphotericin B (≥6 days after diagnosis) was associated with significantly increased mortality as compared with those treated earlier [22]. It is also critical to rapidly identify the risk factors that precipitated the mucormycosis (eg, hyperglycemia, neutropenia, or acidosis) and rapidly correct them.

In this report, we have presented a case of a post-CABG deep sternal wound infection that was ultimately found to be caused by a *Rhizopus* species. This patient was treated with serial debridements, liposomal amphotericin B, and isavuconazonium. Following a prolonged hospitalization, the patient was discharged home in stable condition. Fungal mediastinitis is a rare entity, and physicians must maintain a high index of suspicion when wounds remain persistently infected, persistent tissue necrosis is encountered [23, 24], or routine cultures are negative. This case highlights that a fungal cause of mediastinitis should be suspected in patients with frank deep sternal wound infections, especially in the setting of poorly controlled diabetes despite negative bacterial cultures. Delayed and subacute onset of symptoms often distinguishes fungal mediastinitis from bacterial mediastinitis.

## Notes

***Acknowledgments.*** The authors thank Bashi Ebrahimi, MT (ASCP), for obtaining the tape preparation image used in Figure 2*A* and Brandon Larsen, MD, PhD, for providing the hematoxylin and eosin–stained slide in Figure 2*B*.

***Author contributions.*** C. A. C. and J. E. B. conceived the project. C. A. C. wrote the initial manuscript draft. T. E. G., F. X. D., C. F. S., R. O., and J. E. B. edited the initial manuscript and contributed to the final published manuscript.

***Patient consent statement.*** Written consent for publication was obtained from the patient.

***Funding***. None.
